# Lupus érythémateux systémique révélé par une pneumopathie interstitielle diffuse

**DOI:** 10.11604/pamj.2015.22.80.7787

**Published:** 2015-10-01

**Authors:** Naziha Khammassi, Haykel Abdelhedi

**Affiliations:** 1 Service de Médecine Interne, Hôpital Razi 2010, La Manouba, Tunisie

**Keywords:** Lupus érythémateux systémique, atteinte pulmonaire, pneumopathie interstitielle diffuse, Systemic lupus erythematosus, pulmonary involvement, diffuse interstitial pneumonia

## Image en medicine

Le lupus érythémateux systémique (LES) est une connectivite qui se traduit le plus souvent par une atteinte multiviscérale, il est associé à des manifestations pleuropulmonaires dans plus de 50% des cas. Néanmoins, la pneumopathie interstitielle diffuse (PID) reste une atteinte rare et elle n'est qu'exceptionnellement révélatrice. Patiente âgée de 49 ans, hospitalisée pour exploration d'adénopathies inguinales avec dyspnée d'effort stade II (classification NYHA). L'examen trouvait une patiente en bon état général, eupnéique au repos, des adénopathies jugulo-carotidiennes infracentimétriques bilatérales et des adénopathies inguinales bilatérales. À l'auscultation pulmonaire on notait des râles sous crépitants au niveau des 2 bases pulmonaires. A la biologie il y avait une anémie normochrome normocytaire à 9,8g/dl d'hémoglobine, une lymphopénie à 480/mm^3^ et un syndrome inflammatoire biologique. Le bilan phosphocalcique sanguin et urinaire était normal. La TDM thoraco-abdomino-pelvienne montrait des adénomégalies cervico-thoraco-abdominales, un épanchement péricardique de faible abondance et une PID dont l'aspect évoque une pneumopathie infiltrante lymphocytaire. Le bilan immunologique trouvait des anticorps antinucléaires positifs à 1/3200 et des anticorps anti-DNA natifs positifs. Le diagnostic de LES avec atteinte pulmonaire (PID) a été retenu devant: la péricardite, la lymphopénie, les anticorps antinucléaires et anticorps anti-DNA natifs positifs. Les étiologies des PID subaigües ou chroniques sont dominées par la sarcoïdose, la fibrose pulmonaire idiopathique et les fibroses pulmonaires associées aux connectivites (sclérodermie, syndrome de gougerot sjogren…). Une exploration fonctionnelle respiratoire faite avant l'instauration du traitement était normale et une corticothérapie a été instaurée à la dose de 1mg/kg/j avec une évolution clinique favorable.

**Figure 1:**
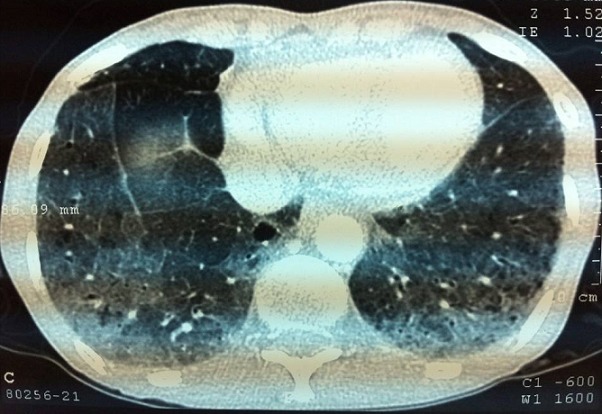
Scanner thoracique: multiples lésions diffuses prédominants au niveau des deux bases. Epaississement des parois bronchiques. Plage d'hyperdensité en verre dépoli

